# Threshold-Switching Memristors for Neuromorphic Thermoreception

**DOI:** 10.3390/s25051533

**Published:** 2025-03-01

**Authors:** Haotian Li, Chunsheng Jiang, Qilin Hua

**Affiliations:** 1 School of Integrated Circuits and Electronics, Beijing Institute of Technology, Beijing 100081, China; 2 Guangxi Key Laboratory of Brain-Inspired Computing and Intelligent Chips, Guangxi Normal University, Guilin 541004, China

**Keywords:** memristor, artificial sensory system, thermoreceptor

## Abstract

Neuromorphic devices emulating the temperature-sensing capabilities of biological thermoreceptors hold significant promise for neuron-like artificial sensory systems. In this work, Bi_2_Se_3_-based threshold-switching memristors were presented in constructing temperature-sensing neuron circuits, leveraging its exceptional attributes, such as high switching ratio (>10^6^), low threshold voltage, and thermoelectric response. The spiking oscillation response of the devices to resistance and temperature variations was analyzed using Hspice simulation of the memristor model based on its resistance in on/off states, threshold voltage (*V_th_*), and hold voltage (*V_hold_*). These results show the great potential of the Bi_2_Se_3_-based memristor in enabling biorealistic thermoreception applications.

## 1. Introduction

As artificial intelligence (AI) computational capabilities surge exponentially in an explosive manner and Moore’s Law moves forward encountering bottlenecks, a discrepancy emerges in the advancement of computational power [1,2,3,4]. Due to the limitations of traditional von Neumann computing architectures, their internal storage units and processors can only interact with each other through logical signals, which results in data redundancy and wasted energy consumption [5,6]. Consequently, the transfer of information via pulsed electrical signals, akin to biological organisms, has garnered significant research attention [7,8]. The construction of traditional bionic systems realizes the simulation of bionic function through the emulation of synaptic characteristics and then the construction of neuronal systems [9,10,11]. As an important self-protection mechanism of organisms, there are fewer artificial sensory systems to realize the function of temperature sensing [12,13,14,15,16]. Among the existing research, the biological neuronal sensory systems exhibit intricate complexities, often entailing challenges inherent to conventional complementary metal–oxide semiconductor (CMOS) device-based implementations [8,17].

Memristive devices, because of their own conductance in relation to the applied stimulus, allow the emulation of synaptic dynamic features at the single device level. In particular, the emerging threshold-switching (TS) behavior of memristors offers a pathway to achieve neuron-like thresholding and excitation behavior within a simplistic circuit structure, necessitating only resistors and capacitors. This efficacy stems from its inherent attributes of resistance switching, simple structure, and low threshold voltage [18,19]. Conventional transition metal–oxide (e.g., HfO_2_)-based memristors are non-volatile devices that find applications in areas such as neural networks, chaos, and nonlinear circuits [6,20,21,22,23,24]. In contrast, volatile threshold-switching devices are commonly utilized in constructing artificial neurons. For instance, the use of Bi_2_Se_3_, a topological insulator material, shows unique thermoelectric sensation and excellent electrical properties (e.g., low leakage current and reduced threshold voltage) [25], facilitating the realization of artificial neuron circuits characterized by minimal power consumption (<1 μW) and rapid switching speeds.

In this work, we outline the preparation of the Bi_2_Se_3_ material, known for its thermoelectric properties, as a functional layer for memristive devices. And Bi_2_Se_3_-based threshold- switching (TS) memristors were designed and fabricated to construct an artificial neuron circuit with thermoreception. The oscillatory output of the circuit was simulated under various conditions, including resistance and temperature variations. Such devices are poised to advance the development of multifunctional neuromorphic sensory systems.

## 2. Material and Methods

**Figure 1:**
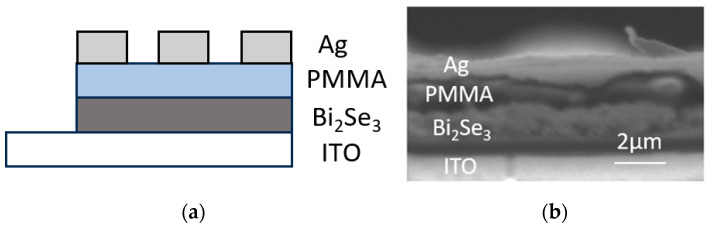
(**a**) Schematic diagram of structure design of the Bi_2_Se_3_-based memristor; (**b**) cross-sectional SEM image of the Bi_2_Se_3_-based memristor.

**Figure 2:**
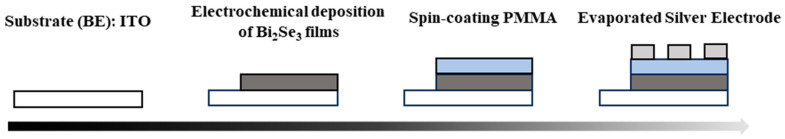
The fabrication process flowchart of the Bi_2_Se_3_-based memristor.

**Figure 3:**
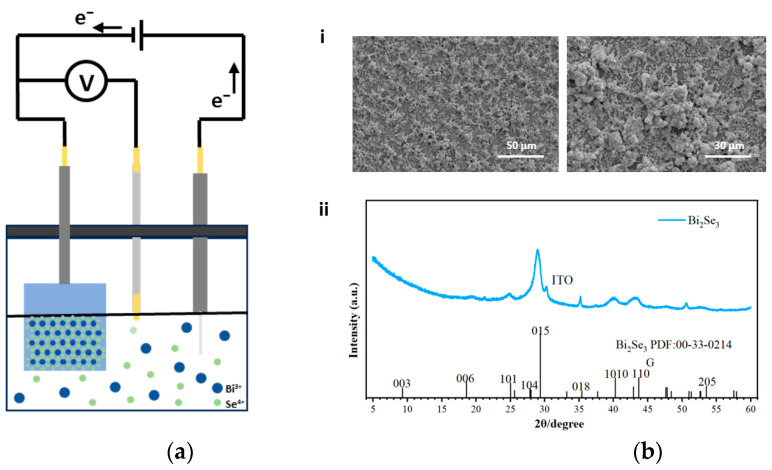
Preparation and characterization of Bi_2_Se_3_ films: (**a**) schematic illustration of the electrochemical deposition of Bi_2_Se_3_ films; (**b**) i: SEM images of deposited Bi_2_Se_3_ film at different magnifications; ii: X-ray diffraction (XRD) pattern of the as-grown Bi_2_Se_3_ film.

The Bi_2_Se_3_-based TS memristor is designed with a stacked structure of ITO/Bi_2_Se_3_/PMMA/Ag, as shown in Figure 1a. The cross-sectional scanning electron microscopy (SEM) image (Figure 1b) clearly demonstrates the pronounced multilayer morphologies. The fabrication process flowchart of the Bi_2_Se_3_-based TS memristor is illustrated in Figure 2. Initially, the Bi_2_Se_3_ thin films were grown on an ITO-film-coated (2.5 × 2.5 cm^2^) glass substrate using the electrochemical deposition method with a three-electrode configuration, as illustrated in Figure 3a.

A solution system comprising 3 mM Bi(NO_3_)_3_∙5H_2_O + 3 mM SeO_2_ was employed; before deposition, the prepared electrolyte solution needed to be evacuated to remove deoxygenation from the solution, and the ITO-film-coated glass substrate was cleaned with acetone, absolute ethanol, and deionized water, respectively. After that, a deposition voltage at −0.4 V was applied for 1500 s to obtain Bi_2_Se_3_ films on the working electrode at 300 K. Given that the surface of the directly electrochemically deposited Bi_2_Se_3_ film is not uniformly smooth, and the material’s low energy level tends to form Ohmic contacts with silver electrodes, a poly methyl methacrylate (PMMA) layer was utilized as a barrier layer. This PMMA layer acts as both an encapsulation to protect the functional layer of Bi_2_Se_3_ and a means to smoothen the material’s surface, facilitating the attachment and contact of the top electrode. Specifically, a dimethylformamide (DMF) solution containing dissolved PMMA was spin-coated onto the dried Bi_2_Se_3_ film at 1200 r/s three times to obtain a slippery PMMA barrier layer surface. Subsequently, an Ag thin film with a top electrode size of 750 μm was fabricated through an evaporation method, and a single-layer thickness of 120 nm served as the top electrode. Finally, the Bi_2_Se_3_-based TS memristor was successfully obtained.

In the electrochemical deposition system, as depicted in Figure 3a, we employed a calomel electrode as the reference electrode, a platinum wire electrode as the counter electrode, and the ITO conductive glass as the working electrode. Subsequently, Bi_2_Se_3_ films were deposited by applying external potentials between the working and counter electrodes. To assess the quality of the deposited Bi_2_Se_3_ films, the surface morphology and material composition were characterized using scanning electron microscopy (SEM) and X-ray diffraction (XRD), respectively, as illustrated in Figure 3b. The SEM images show that the deposited Bi_2_Se_3_ material effectively adheres to the ITO surface. Moreover, the XRD result reveals prominent diffraction peaks, indicating the high crystallinity and sizable grain size of the Bi_2_Se_3_ films achievable through electrochemical deposition.

## 3. Results

### 3.1. Device Performance

**Figure 4:**
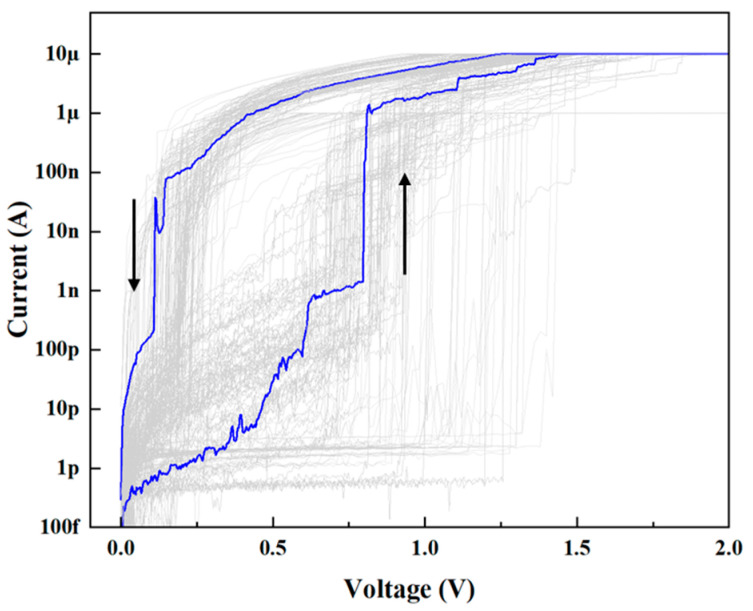
Cyclic I–V characteristics of the Bi_2_Se_3_-based memristor (The arrow indicates the direction of the I–V sweeping).

To evaluate the switching performance of the Bi_2_Se_3_-based TS memristor, the device was subjected to current–voltage (I–V) sweeping at a compliant current of 10 μA. Figure 4 shows the I–V curves of the device, with the arrows indicating the direction of resistance transition, exhibiting pronounced volatile threshold-switching behavior. Under the current compliance of 10 μA, the device switches from the high resistance state (HRS) to the low resistance state (LRS) at a threshold voltage (*V_th_*) between 0.6 V and 1.5 V, and then, the device switches from the LRS to the HRS when the voltage retraces to a hold voltage (*V_hold_*) of around 0.2 V. Meanwhile, the device is able to maintain its LRS throughout the continuous sweeping process and return to its initial HRS when the applied voltage is removed. Even after more than 100 cycles, the device exhibits stable switching behavior.

**Figure 5:**
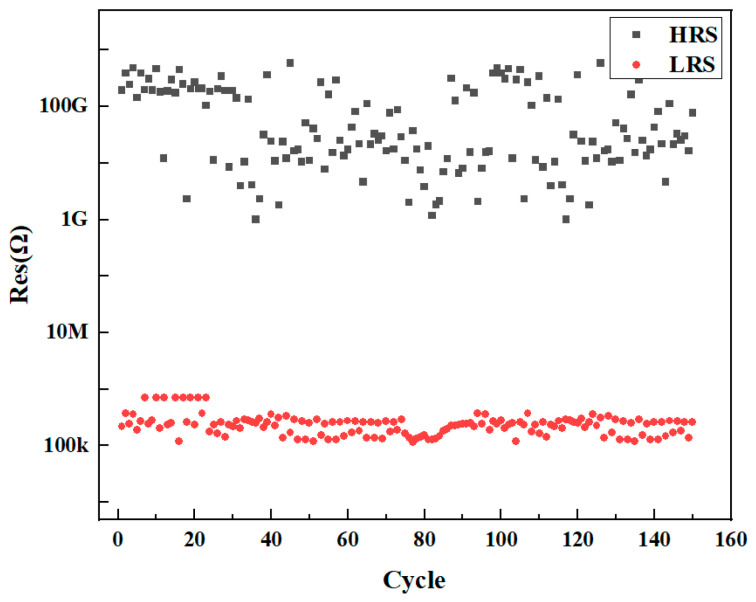
Endurance of the Bi_2_Se_3_-based memristor at a read voltage of 0.1 V with a compliance current (*I_cc_*) of 10 μA.

Additionally, 150 electrical pulses were performed on the device; we recorded the resistance values of the device in the form of electrical pulses during a single cycle, and the HRS and LRS values of the device were derived from the switching of 150 cycles. As shown in Figure 5, it can be seen that the device has a uniform distribution of the HRS and the LRS, showing good stability performance.

**Figure 6:**
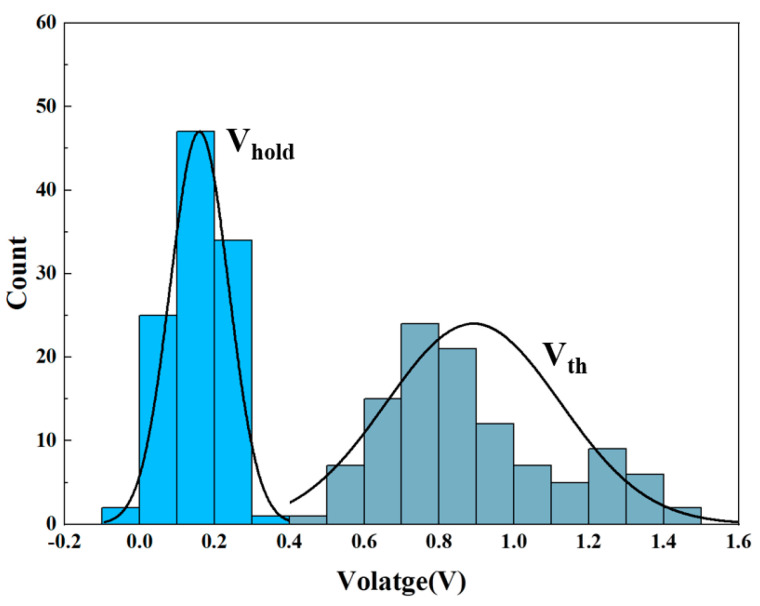
Probability distribution of *V_th_* and *V_hold_* in the Bi_2_Se_3_-based memristor.

Figure 6 shows the correspondence of device measurement data for a volatile threshold-switching device over 150 forward sweep voltage cycles, including the *V_th_* and *V_hold_*. The *V_th_* and *V_hold_* of the device were statistically analyzed, and it was found that the *Vth* distribution of the device was more dispersed at 0.9 V, while the *V_hold_* distribution was stable at 0.2 V, but all of them tended to converge with the normal distribution in a range, which demonstrates good cycling stability of the device. 

**Figure 7:**
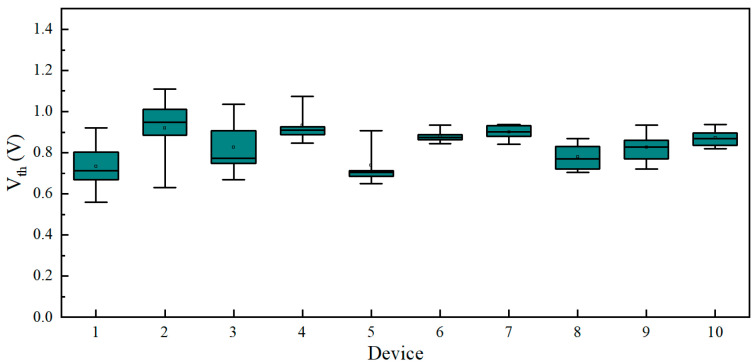
Statistical distribution of threshold voltages for 10 devices.

To assess device-to-device variability, we further conducted I–V characteristic tests on ten devices on a single substrate, recording their threshold voltages. Each device was subjected to ten consecutive operations. As illustrated in Figure 7, the Vth of the memristor falls within the range of 0.6 V to 1.2 V, indicating comparable uniformity.

**Figure 8:**
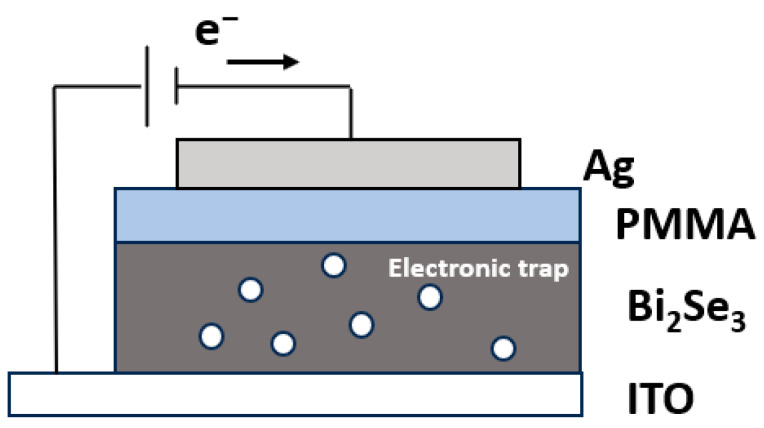
Schematic diagram of the resistance switching mechanism of the Bi_2_Se_3_-based memristor.

Based on the I–V characteristics of the device and the material properties of Bi_2_Se_3_, we employ a charge-trap model to elucidate the electron trapping and releasing process that underlies the resistive switching mechanism of the memristor, as shown in Figure 8. In instances where the device initiates in an intermediate resistance state, the application of a bias voltage would expel trapped electrons, activating a greater number of traps to effectuate the transition to the HRS. Upon the application of the activation voltage, the device reverts to the HRS state. During the subsequent scanning process of applying the forward sweep voltage from 0 V to 2 V, the device undergoes a switching transition from the HRS to the LRS as electrons from the top electrode traverse the PMMA barrier layer, filling up the electron traps. As the electron trap becomes saturated with electrons, the injected carriers gain mobility within the dielectric layer, leading to the device exhibiting a switch from the HRS to the LRS.

Throughout the continuous process of applying the forward sweeping voltage, the device remains in the LRS until the applied forward voltage drops below the *V_hold_*, characteristic of a threshold-type switching device. Specifically, the resistance switching behavior manifests as the forward sweep voltage scan decreases from 2 V to 0.2 V, causing the detached electrons from the trap to transition the device back from the LRS to the HRS. Consequently, upon the conclusion of the forward voltage application, the device returns to the HRS. This reversible process, as illustrated in Figure 8, denotes the volatile memristive characteristics.

**Figure 9:**
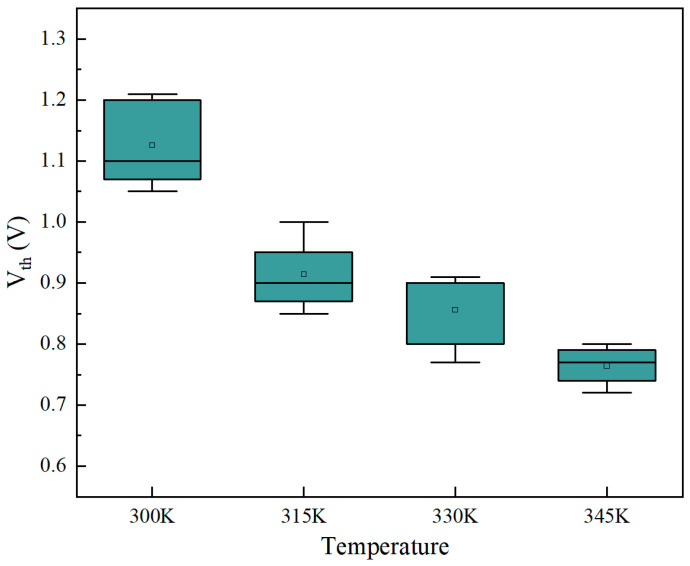
*V_th_* of the Bi_2_Se_3_-based memristor under different temperatures (at 300 K, 315 K, 330 K, and 345 K).

To validate the temperature-sensing capabilities of the Bi_2_Se_3_-based memristor, a thermal test platform equipped with a chuck was constructed to analyze the variation in threshold voltage across different temperatures. Initially, the memristor device was configured to the HRS and subjected to a temperature increment from room temperature (300 K) to 345 K. At intervals of 15 K, I–V scans were conducted on the device to monitor its temperature-dependent output response. The output relationship between the device and the temperature response is shown in Figure 9. We conducted tests on the device through five cyclic I–V scans at varying temperatures, ranging from 300 K to 345 K. Observing a decrease of approximately 0.4 V in the device’s threshold voltage with rising temperatures. Subsequently, we incorporated the average values from the box-plot analysis at different temperatures into the device model for circuit simulation, affirming the device’s potential suitability for application in neuromorphic circuits.

In order to provide a comprehensive characterization of the Bi_2_Se_3_-based memristor, we have conducted a comparative analysis of the device parameters with findings from previous studies on volatile memristive devices [22,23,24,25]. Table 1 illustrates that the Bi_2_Se_3_-based memristor has comparable performance to the typical devices, in terms of operating voltage (*V_th_*), ON/OFF ratio, and energy consumption per switching event (expressed as leakage current (*I_off_*)). Additionally, when compared to planar devices, the Bi_2_Se_3_-based memristor exhibits enhanced cycling stability due to its stacked devices. Functioning as a temperature-sensitive switching threshold device, the Bi_2_Se_3_-based memristor developed in this work demonstrates a lower leakage current and a higher ON/OFF ratio. These attributes signify substantial potential for the implementation of memristor-based neurons with temperature-sensing capabilities in an ultralow-power fashion. Subsequently, we intend to delve deeper into the validation of incorporating this device into neuronal circuits through simulation.

### 3.2. Neuronal Circuit Simulation

**Figure 10:**
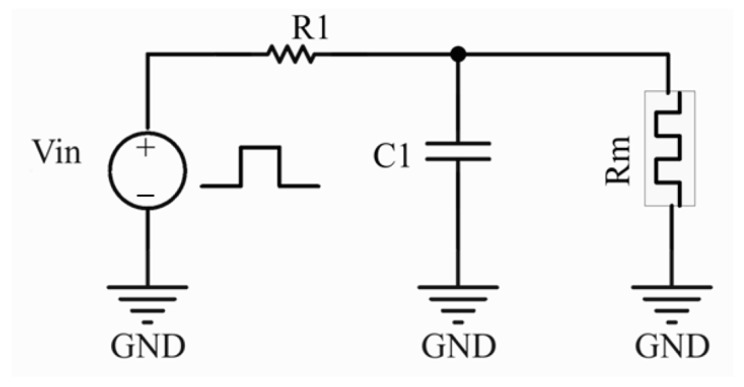
Schematic diagram of the constructed artificial neuron circuit (i.e., thermoreceptor).

Due to the unavoidable system complexity of neuronal circuits based on CMOS devices, we constructed artificial neuronal circuits with a simple structure based on the memristor neuronal oscillatory function. And the feasibility of the device for neuronal circuits is further investigated through circuit simulations. The TS behavior of the device, transitioning between HRS and LRS, can be roughly equated to a “two-resistor model”, where HRS and LRS are represented as two constant resistors [29]. Based on this, we built a neuronal circuit consisting of multiple passive devices by means of a neuronal-functioning memristive device, in which the memristive device performs a function similar to neuronal leakage integration and excitation. In detail, a memristor model is established based on empirical data. At 300 K, the memristor exhibits a *V_th_* of 1.1 V, *V_hold_* of 0.2 V, HRS at 100 GΩ, and the LRS is 100 kΩ. Subsequently, a neuronal circuit is devised utilizing the memristive device, as shown in Figure 10, comprising an RC oscillator circuit and a voltage divider circuit. 

In the RC oscillator circuit, the output voltage frequency can be controlled by changing the resistance value of *R*_1_ in the circuit. Firstly, the *t_rise_* of the output voltage is calculated by the following equation:

*t_rise_* = *R_eq_C*_1_*ln*[(*V_in_/R*_1_ − *V_hold_*/*R_eq_*)/(*V_in_/R*_1_ − *V_th_/R_eq_*)] (1)

In Equation (1), *R_off_* stands for the resistance value of the HRS of the memristor, and *V_th_* and *V_hold_* represent the threshold and hold voltages of the memristor, respectively. *R*_1_ denotes the resistance value in the oscillator circuit. When *R_off_* ≫ *R*_1_, *R_eq_* = *R_off_* ‖ *R*_1_ ≈ *R*_1_, *R_eq_* can be approximated as *R*_1_, and *f* can be regarded as primarily affected by *R*_1_.

Above all, the frequency is determined by the following equation:

*f* ∝ 1/*t_rise_* ≈ 1/*R*_1_*C*_1_*ln*[(*V_in_* − *V_hold_*)/(*V_in_* − *V_th_*)] (2)

**Figure 11:**
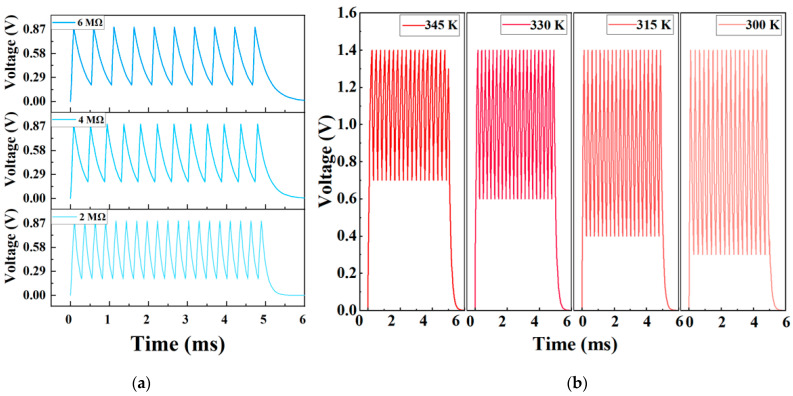
Impulse simulation response of neuronal circuits: (**a**) simulated oscillation characteristics of the thermoreceptor in response to various values of *R*_1_ in the neuron circuit (2 MΩ, 4 MΩ, and 6 MΩ); (**b**) simulated oscillation characteristics of the thermoreceptor in response to various *V_th_* (0.8 V, 0.9 V, 1.1 V, and 1.2 V) of Bi_2_Se_3_-based memristor under different temperatures.

Following the construction of the artificial neuron simulation model, the feasibility of the memristive device for developing artificial neuron circuits is verified by changing the value of *R*_1_ and the model parameters of the memristor in the circuit, respectively. By observing the resulting alterations in the output signal of the oscillator circuit, the efficacy of the memristive device in artificial neuron circuitry is confirmed. Initially, we varied the resistor values (R_1_: 2 MΩ, 4 MΩ, and 6 MΩ) in the RC circuit. In response to the same pulse voltage, as shown in Figure 11a, the output voltage frequency (*f*) shifted from 4 kHz to 2 kHz with the increasing resistance value of *R*_1_, while the peak value remained relatively stable. By substituting *R*_1_ with a resistive sensor, the circuit can effectively convey external information via a frequency signal, enabling the neuronal circuitry to sense external environmental signals.

Subsequently, to further assess the impact of temperature on the output signal of the neuronal circuit, we introduced various *V_th_* values at different temperatures (Figure 9) into the memristor model, as shown in Figure 11b. Upon observing temperature fluctuations (i.e., 300 K, 315 K, 330 K, and 345 K), the peak-to-peak output voltage ranges from 1.1 V to 0.7 V, while the frequency exhibited minimal change. Consequently, the potential application of this neuron circuit in multimodal sensing circuits shows promise in conveying information through the peak and frequency of the output signal. This capability facilitates the realization of artificial thermoreception, which can be leveraged for multimodal spiking-based sensing of the external environment.

## 4. Conclusions

In summary, drawing inspiration from the temperature-sensing mechanisms in living organisms [30,31], we successfully fabricated threshold-switching memristors based on Bi_2_Se_3_ material through the electrochemical deposition method. The device demonstrates the ability to switch between the HRS and LRS attributed to the function of the PMMA layer barrier layer and the charge trapping and de-trapping function of the Bi_2_Se_3_ functional layer. Specifically, we have substantiated the device’s performance, including high switching ratio (>10^6^), low threshold voltage, high endurance, and temperature sensation through I–V cycling tests. A benefit of the thermoelectric properties of the Bi_2_Se_3_ material itself, the Bi_2_Se_3_-based memristor exhibits temperature-sensing capability. Performance testing at different temperatures (300 K, 315 K, 330 K, and 345 K) further confirms this feature. Leveraging the Bi_2_Se_3_-based memristor model at different temperatures in neuronal circuit simulations allows us to unlock the device’s potential for neuronal functionalities such as oscillation and frequency response. The feasibility of neuronal circuits based on Bi_2_Se_3_ material for artificial thermoreceptors was further shown. Notably, this work exhibits immense promise for advancing biorealistic thermoreceptors and multimodal neuromorphic sensory systems, and the constructed perceptual neuronal circuits based on Bi_2_Se_3_ material could be used in edge devices for human–computer interaction [32,33].

## Figures and Tables

**Table 1:** Comparison of the ON/OFF ratio, SET voltage (*V_th_*), leakage current (*I_cc_*), and functions of the Bi_2_Se_3_-based memristor developed in this study with some perceptive planar memristors.

Device	ON/OFF Ratio	*V_th_* (V)	*I_cc_* (A)	Functions
ITO/Bi_2_Se_3_/PMMA/Ag	10^6^	1	10^−9^	Thermoreception
Ag/Ag−In−Zn−S/silk sericin/W [26]	10^2^	0.4	10^−7^	Humidity Sensor
Ag/ZnO/Pt [27]	4 × 10^2^	1.2	10^−7^	Nociceptor
Pt/SiOx:Ag/Ag/Pt [13]	10^7^	0.2	10^−12^	Nociceptor
Ag/HfOx/ITO/PET [28]	10^2^	0.75	10^−4^	Nociceptor

## Data Availability

Data are contained within the article.

## Abbreviations

The following abbreviations are used in this manuscript:

CMOS	Complementary Metal–Oxide Semiconductor
TS	Threshold Switching
PMMA	Poly Methyl Meth-Acrylate
DMF	Dimethylformamide
BE	Bottom Electrode
TE	Top Electrode
SEM	Scanning Electron Microscopy
XRD	X-Ray Diffraction
ITO	Indium Tin Oxide
LRS	Low Resistance State
HRS	High Resistance State
RC	Resistor Capacitance
